# Patient Pain and Satisfaction With 10, 30, and 70 mL Transcervical Foley Balloons for Cervical Ripening During Induction of Labor

**DOI:** 10.7759/cureus.41535

**Published:** 2023-07-07

**Authors:** Inessa Dombrovsky, Kristina Roloff, C. Camille Okekpe, Robert Stowe, Guillermo J Valenzuela

**Affiliations:** 1 Department of Women's Health, Arrowhead Regional Medical Center, Colton, USA

**Keywords:** pregnancy induction, pregnancy, labor and delivery, cervical ripening, time to delivery, oxytocin, satisfaction, pain, transcervical foley catheter, induction of labor

## Abstract

Objective

To assess patient pain and satisfaction and time to delivery following transcervical Foley catheter balloon inflation to 10, 30, or 70 mL with simultaneous administration of oxytocin.

Methods

We performed a randomized prospective study with 30 or 70 mL transcervical Foley balloon catheters in combination with oxytocin during labor induction at term. A 10 mL group was included as a sham control group. Time to delivery was measured, and a patient questionnaire was administered at the time the catheter was expelled to determine patient pain and satisfaction.

Results

In 120 enrolled patients, there was a non-significant trend toward reduced time to delivery in the large Foley balloon group (10 mL: 30:45 ± 38:53, 30 mL: 26:41 ± 20:53, and 70 mL 22:40 ± 15:35, hh:mm, P = 0.412). The pain score at the time the balloon was expelled was significantly higher in the 70 ml group compared to the 10 ml and 30 ml groups (P = 0.004 and P = 0.034, respectively). We found no other differences in patient satisfaction or pain scores at the time of placement of the Foley catheter for the three groups.

Conclusion

Small gains in time to delivery should be balanced against patient experiences, and expectations of pain during the ripening process should be addressed at the time of Foley insertion.

## Introduction

The number of patients undergoing labor induction has increased significantly since the ARRIVE trial was published in 2018, which showed that gestational age of 39 0/7 to 39 6/7 weeks is the optimal time to deliver to reduce maternal and neonatal complications in low-risk pregnancies [1]. Since then, many labor & delivery units have noted increased patient volumes, longer hospital stays, the need for additional nursing staff, and increased costs [2,3]. This has led induction practices to focus on reductions in time to deliver to reduce operational burdens [4]. However, there is a paucity of studies that evaluate patient experiences with pain and satisfaction during induction and specifically with agents that may reduce time to delivery.

Transcervical Foley catheter placement with simultaneous administration of oxytocin or prostaglandins has been shown to result in a shortened time to delivery when compared to the sequential use of cervical ripening agents [3,5,6]. Larger transcervical Foley catheters alone (without concomitant use of prostaglandins or oxytocin) have also been shown to decrease the time to deliver [7-9].

In this study, we assessed patient pain and satisfaction as well as time to delivery following transcervical Foley catheter balloon inflation to 10, 30, or 70 mL with simultaneous administration of oxytocin. We hypothesized we would find shorter times to delivery but higher pain scores with larger balloon volumes.

## Materials and methods

This randomized prospective trial was performed at a single community hospital in San Bernardino, CA. Participants were at least 21 years of age with a term (defined as 37 0/7 or more weeks of gestation) singleton pregnancy in a cephalic position. Patients were induced electively or for medical or obstetric indications and were required to have intact membranes with a Bishop score of six or less. Patients were excluded if there was a contraindication to vaginal delivery, a prior cesarean delivery, a contraindication to transcervical Foley placement (such as unexplained vaginal bleeding, latex allergy, or low-lying placenta), known uterine or vaginal infection, ruptured membranes, multiple gestations, or if the patient did not read in either English or Spanish, or was unable or unwilling to give informed consent.

Patients who consented were randomized in blocks controlled for parity to determine balloon volume. Foley catheters designed for 10, 30, or 70 mL balloon volumes were used, and no balloons were over-inflated. The 10 mL group was included to simulate a sham control. All patients received simultaneous oxytocin administration upon placement of the catheter using a standard low-dose protocol beginning with 2 milliunits/minute and increasing by 1-2 milliunits/minute every 30 minutes until the patient achieved four to five strong contractions over a 10-minute period. The Foley catheter was left in place until expelled or removed if cesarean delivery was indicated. Participants were excluded if any other cervical ripening agents were used prior to the insertion of the catheter. When the Foley catheter was expelled, the patient was given a questionnaire assessing their pain and satisfaction with the labor induction. Management of the induction following expulsion or removal of the Foley catheter was carried out at the discretion of the attending physician and additional ripening agents may have been administered.

Maternal satisfaction and pain were assessed through a written questionnaire. To assess the patient experience due to transcervical Foley in combination with IV oxytocin and no other agents, the questionnaire was administered at the time of Foley expulsion or removal. Cronbach's alpha was calculated to determine the internal reliability of the scale for two domains: pain and satisfaction. Questions determined to be misinterpreted were discarded. Results of the survey were compared for the three groups using a one-way ANOVA followed by a least squared difference test.

The length of time to delivery was defined as the duration from Foley catheter placement to birth time regardless of type of delivery (cesarean or vaginal), in an intent-to-treat analysis. Other collected measures included BMI, race, type of delivery, use of intravenous pain medication or epidural, maximum dose of oxytocin administered, presence of chorioamnionitis, presence of postpartum hemorrhage, APGAR (appearance, pulse, grimace, activity, and respiration) scores, neonatal blood gases, and NICU admission. Ordinal and continuous variables were compared for the three groups using a one-way ANOVA. The least squared difference test was used to determine which groups differed if a statistically significant difference was noted. Categorical variables were compared using a chi-squared test. Univariate ANOVA was used to determine the interaction effects of significant differences in baseline characteristics.

Prior published literature comparing balloon volumes and time to delivery without other induction agents was used for sample size estimates. Our institution’s mean time to delivery is approximately 24 hours (±11 hours) from the initiation of induction [10]. To test the null hypothesis that the mean time to delivery was less than four hours different between groups (20, 24, and 28 hours to delivery) in a one-way ANOVA with 5% alpha error, 50% beta error, and 80% power, the sample size would be 38 patients per arm (114 patients total). To detect a six-hour difference (18, 24, and 30 hours, respectively), only 18 patients per arm would be needed. Differences smaller than four hours between groups were considered clinically insignificant. This sample size was not sufficient to determine intergroup differences or perform multiple comparisons.

Statistical analysis was performed using SPSS (version 27.0.0.0, IBM Corp., Armonk, NY), and statistical significance was considered at a P = 0.05. This study was conducted with approval from the Arrowhead Regional Medical Center Institutional Review Board (Protocol #: 20-14). Written informed consent was obtained from all participants prior to randomization.

## Results

A total of 120 patients were recruited. Forty were randomized to each balloon volume, i.e., 10, 30, or 70 mL. Because of the blocked randomization scheme to control for parity, exactly 60 (50%) of the patients were nulliparous at randomization. Demographics of the sample are shown in Table 1.

**Table 1:** Demographic characteristics. Data are mean ± standard deviation or N (%) where appropriate. Totals may not add up to 100% due to missing data or rounding errors.

	Mean or N		Range
Age (years)	25.8	(±5.4)	21-45
Gravidity	2.48	(±1.6)	1-7
Parity	0.94	(±1.1)	0-5
Gestational age (weeks)	39.1	(±0.9)	37.0-41.1
BMI (kg/m2)	33.48	(±6.6)	20-50
Bishop score	2.67	(±1.6)	0-6
Race			
Hispanic	100	(83.3%)	
Black	6	(5.0%)	
White	13	(10.8%)	
Other	1	(0.08%)	

A total of 105 (88%) of the patients completed a questionnaire at the time the Foley catheter was expelled (the survey is available in the Appendix). Cronbach's alpha for the five survey questions regarding satisfaction was 0.736, and for the three questions regarding pain during the procedure was 0.557. Cronbach's alpha increased to 0.685 if question 4 “I experienced pain during labor” was removed. As the questionnaire was administered at the time of expulsion or removal of the Foley catheter and prior to the onset of labor, there is a high probability this question was difficult to understand and was therefore discarded.

The mean pain score at the time the Foley catheter was expelled was significantly higher in the 70 mL group as compared to the 10 mL or 30 mL groups (P = 0.035, 70 mL compared to 10 mL P = 0.004, 70 mL compared to 30 mL P = 0.034) (Table 2).

**Table 2:** Demographics, labor characteristics, and delivery times for the three transcervical Foley catheter size groups. Data are mean ± standard deviation or N (%) where appropriate. Totals may not add up to 100% due to missing data or rounding errors.

	10 mL		30 mL		70 mL		P
	(N = 40)		(N = 40)		(N = 40)		
Age (years)	25.2	(±5.3)	26.4	(±5.6)	25.7	(±5.2)	0.621
Gravidity	2.4	(±1.5)	2.4	(±1.7)	2.5	(±1.5)	0.948
Parity	0.93	(±1.1)	0.93	(±1.1)	0.98	(±1.1)	0.975
Gestational age	39.2	(±0.8)	39.1	(±1.1)	38.9	(±0.8)	0.330
BMI (kg/m2)	33.1	(±7.1)	33.3	(±5.4)	34.0	(±7.2)	0.809
Nulliparous	20	(50%)	20	(50%)	20	(50%)	1.00
Multiparous	20	(50%)	20	(50%)	20	(50%)	
BMI category							
Not obese	10	(25.0%)	12	(30%)	10	(25.0%)	0.952
Class 1 obesity (BMI = 30-34.9)	15	(37.5%)	14	(35.0%)	13	(32.5%)	
Class 2 obesity (BMI = 35-39.9)	8	(20.0%)	8	(20.0%)	7	(17.5%)	
Class 3 obesity (BMI >= 40)	7	(17.5%)	6	(15.0%)	10	(25.0%)	
Race							
Hispanic	32	(80%)	32	(80%)	36	(90.0%)	0.644
Black	4	(10%)	2	(5%)	1	(2.5%)	
White	4	(10%)	6	(15%)	3	(7.5%)	
Labor characteristics							
Bishop score (placement)	3.0	(±1.5)	1.9	(±1.6)	3.1	(±1.5)	0.003
Bishop score (removal)	4.8	(±2.2)	4.9	(±1.9)	6.0	(±1.7)	0.010
Type of pain control							0.565
None	3	(7.5%)	5	(12.5%)	3	(7.5%)	
Narcotics (IV Stadol)	2	(5.0%)	2	(5.0%)	0		
Epidural	35	(87.5%)	33	(82.5%)	37	(92.5%)	
Maximum oxytocin infusion rate (milliunits/min)	13.3	(±5.1)	13.9	(±6.2)	13.2	(±6.2)	0.836
Duration of induction to delivery							
Time Foley in place (h:mm)	3:14	(±2:44)	4:24	(±2:23)	6:10	(±4:44)	0.001
Time Foley placement to delivery (h:mm)	30:45	(±38:53)	26:41	(±20:53)	22:40	(±15:35)	0.412
Mode of delivery							
Vaginal delivery	31	(77.5%)	32	(80%)	34	(85%)	0.887
Vacuum-assisted vaginal delivery	3	(7.5%)	3	(7.5%)	3	(7.5%)	
Cesarean delivery	6	(15%)	5	(12.5%)	3	(7.5%)	
Foley pain score							
Pain at Foley placement	3.98	(±2.61)	3.79	(±2.69)	4.08	(±3.06)	0.903
Pain at Foley removal	2.33	(±2.62)	2.64	(±2.71)	3.90	(±3.07)	0.035
Complications							
Chorioamnionitis	3	(7.5%)	2	(5.0%)	5	(12.5%)	0.446
Postpartum hemorrhage	3	(7.5%)	2	(5.0%)	4	(10.0%)	0.697
NICU admission	1	(2.5%)	4	(10.0%)	2	(5.0%)	0.346
Low Apgar at 1 minute	3	(7.5%)	1	(2.5%)	0		0.164
Low Apgar at 5 minutes	1	(2.5%)	0		0		0.365
Low neonatal cord pH	0		0		0		N/A
High neonatal cord base excess	2	(5.1%)	1	(2.5%)	1	(2.5%)	0.757

**Figure 1:**
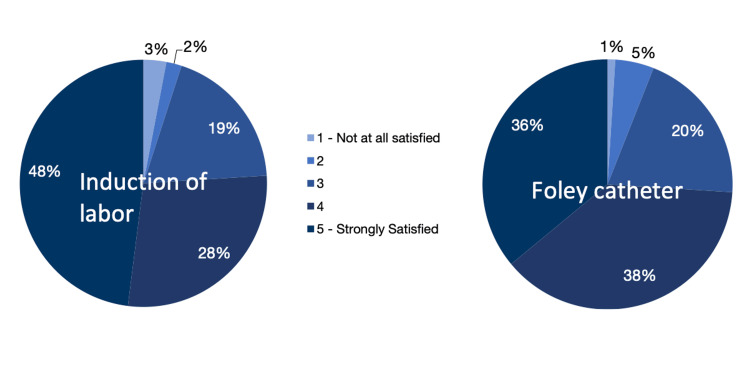
Pie graph showing patient satisfaction answers on a five-point Likert scale for induction of labor and for Foley catheter use as part of induction of labor. A large majority of patients were satisfied with both.

However, there were no differences in pain scores during Foley expulsion between the 30 mL group and the sham 10 mL group (P = 0.456). There was no difference in pain scores at the time of insertion of the Foley catheter. Patient satisfaction with Foley catheter induction was very high and did not differ between the three groups (Table 3 and Figure 1). No patients requested early removal of the Foley catheter for discomfort.

**Table 3:** Results of the patient survey regarding satisfaction and perception of pain. Data are mean ± standard deviation. Survey responses were measured on a Likert scale from 1, strongly disagree to 5, strongly agree, or 1, not at all satisfied to 5, strongly satisfied.

	10 mL		30 mL		70 mL		P
	(N = 37)		(N = 35)		(N = 34)		
I felt well-informed about the method of induction that my provider would be using	4.62	(±0.639)	4.66	(±0.539)	4.65	(±0.485)	0.962
I experienced pain during the placement of the Foley catheter	3.59	(±1.235)	3.46	(±0.980)	3.56	(±1.160)	0.868
I experienced pain while the Foley catheter was in place	2.38	(±1.233)	2.74	(±1.094)	3.03	(±1.337)	0.085
I experienced pain during the labor	3.46	(±0.989)	3.23	(±1.031)	3.00	(±1.015)	0.166
I am satisfied with the treatment	4.16	(±0.928)	4.14	(±0.845)	3.85	(±0.925)	0.281
I would recommend this method of induction to a friend or family member	3.62	(±1.255)	3.86	(±0.944)	3.65	(±0.917)	0.590
How satisfied are you with your method of induction?	4.11	(±1.197)	4.26	(±0.817)	4.15	(±0.925)	0.808
How satisfied are you with your overall treatment?	4.43	(±0.835)	4.57	(±0.655)	4.44	(±0.824)	0.702

We found no difference in pain management between the three groups (P = 0.565; Table 2). One patient randomized to the 70 mL balloon received an epidural prior to the insertion of the Foley. Two patients (one in the 10 mL and one in the 30 mL group) received an epidural while the Foley catheter was still in place. Because epidural use was infrequent during the ripening process, it should not have affected overall pain scores.

We noted a trend toward shorter delivery times in the 70 mL catheter group, but this did not reach statistical significance (10 mL: 30:45 ± 38:53, 30 mL: 26:41 ± 20:53, and 70 mL 22:40 ± 15:35, hh:mm, P = 0.412). The 10 mL balloon remained in place for a significantly shorter duration of time compared to the 70 mL balloon (10 mL: 03:14 ± 02:44; 70 mL: 06:10 ± 04:44 hh:mm, P = 0.001) (Table 2). The pooled standard deviation in this sample was much longer (27 hours) than anticipated from previous studies at our institution. Thus, this sample size is only powered to detect a nine-hour difference between groups.

The starting Bishop score of patients in the 30 mL balloon group (1.9 ± 1.6) was lower than compared to the 10 mL group (3.0 ± 1.5, P = 0.003), and 70 mL group (3.1 ± 1.5, P = 0.003) (Table 2). Univariate ANOVA for time to delivery based on balloon volume controlled for Bishop score showed no significant difference in nulliparous or multiparous patients and no interaction between balloon volume and Bishop score (P = 0.497 nulliparous, P = 0.628 multiparous). Thus, the lack of difference in time to delivery between the three groups was not due to differences in the starting Bishop scores.

We found no difference in demographic characteristics, Bishop score at the time of catheter expulsion, type of pain control used, maximum oxytocin infusion rate, or type of delivery between the three groups. The cesarean delivery rate was low at 11%. There were no differences in maternal or neonatal complications (Table 2).

## Discussion

Patients had significantly higher pain scores when the catheter was expelled with 70 mL transcervical Foley catheter balloons. Overall, we noted high patient satisfaction scores, and no differences in pain at the time of placement of the catheter, regardless of the size of the catheter used. We found a trend toward shorter delivery times with the 70 mL transcervical Foley catheter though the 70 mL balloon stayed in place longer, with approximately four hours difference between groups. Unfortunately, due to an unexpectedly wider standard deviation of the pooled time to delivery for this sample, we were only powered to detect a nine-hour difference between groups with this sample size.

In practice, Foley balloon catheters are often overinflated to accommodate larger volumes, which leads to balloon rupture in approximately 0.9% of cases [11]. We did not experience any balloon ruptures, probably because we used larger balloon volumes rather than over-inflating smaller balloons. Our results suggest that the volume of the balloon does not markedly affect the total time to delivery, and the practice of over-inflating to high volumes is unnecessary, especially given the higher pain scores seen when the 70 mL balloon is expelled.

Prior studies comparing Foley balloon volumes and time to delivery often do not standardize the use of other cervical ripening agents or administer oxytocin simultaneously [8,9,12-15]. Because of their larger size, higher volume Foley balloons, when used for cervical ripening, have been associated with higher Bishop scores when expelled, but not necessarily reductions in time to delivery [16]. Delaney et al. showed that a Foley balloon inflated to 60 mL was associated with a higher proportion of women achieving delivery within 12 hours [7]. However, this study was conducted using a single balloon volume size that was overinflated to varying volumes and some study participants received prostaglandins prior to catheter placement, which may have impacted results [7]. Two meta-analyses have shown that women randomized to larger balloon volumes had significantly shorter time from induction to delivery with no differences in cesarean section rate or chorioamnionitis between the groups [16,17]. However, in all the included trials, induction protocols were not standardized, and many additional cervical ripening agents were used sequentially or in combination with transcervical Foley balloons [16,17]. A strength of this study is that transcervical Foley catheters were used in combination with oxytocin, as dual therapy has been shown to reduce time to delivery over a single method alone. Standardization of all participants to receive intravenous oxytocin simultaneously with Foley balloon placement differs from prior studies and allows for a more accurate comparison of balloon sizes and time to delivery. While transcervical Foley catheters in combination with either misoprostol or oxytocin have been shown to be effective at reducing time to delivery, we chose to only use oxytocin here. Future research should compare different balloon volumes with simultaneous administration of misoprostol.

A weakness of this study is the sample size. A larger sample size may be able to determine smaller differences in time to delivery with different catheter sizes. Also, despite randomization, we ended up with differences in starting Bishop scores amongst the groups; however, as described above, these differences do not appear to have affected the time to delivery.

A strength of this study is the incorporation of patient pain and satisfaction assessment. There is a paucity of published information about patient satisfaction with various balloon sizes. Levy et al. found no significant difference between 30 mL and 60 mL groups [9]. Here, we administered a patient questionnaire at the time the catheter was expelled and thus participant experiences could be attributed to cervix ripening with a transcervical Foley in combination with oxytocin as opposed to the labor or delivery experience. We confirmed that Foley balloons are well tolerated with high patient satisfaction scores, but not surprisingly, the largest balloon volume caused more pain at the time of expulsion than both smaller balloon volumes. We found a 30 mL transcervical Foley balloon volume did not increase pain significantly, even during expulsion when compared to a 10 mL sham group.

## Conclusions

A common practice is to inflate a transcervical Foley balloon to the patient’s comfort level at the time of insertion. However, this does not account for pain later in the process as the catheter is expulsed. Small gains in time to delivery should be balanced against patient experiences, and expectations of pain during the ripening process should be addressed at the time of Foley insertion.

## Appendices

Patient survey

To be administered at the time of Foley catheter removal.

Please answer the following questions on a scale of 1-5 with 1 being strongly disagree and 5 being strongly agree.

1. I felt well-informed about the method of induction that my provider would be using.

1. Strongly disagree

2. Disagree

3. Neutral

4. Agree

5. Strongly Agree

2. I experienced pain during the placement of the Foley catheter.

1. Strongly disagree

2. Disagree

3. Neutral

4. Agree

5. Strongly Agree

3. I experienced pain while the Foley catheter was in place.

1. Strongly disagree

2. Disagree

3. Neutral

4. Agree

5. Strongly Agree

4. I experienced pain during labor.

1. Strongly disagree

2. Disagree

3. Neutral

4. Agree

5. Strongly Agree

5. I am satisfied with the treatment.

1. Strongly disagree

2. Disagree

3. Neutral

4. Agree

5. Strongly Agree

6. I would recommend this method of induction to a friend or family member.

1. Strongly disagree

2. Disagree

3. Neutral

4. Agree

5. Strongly Agree

Please answer the following on a scale of 1-5 with 1 being not at all and 5 being extremely satisfied.

1. How satisfied are you with your method of induction?

1. Not at all satisfied

2. Somewhat dissatisfied

3. Neutral

4. Somewhat satisfied

5. Strongly satisfied

2. How satisfied are you with your overall treatment?

1. Not at all satisfied

2. Somewhat dissatisfied

3. Neutral

4. Somewhat satisfied

5. Strongly satisfied

Please answer the following questions with yes or no.

1. I had an epidural before my Foley catheter was placed. - YES, NO

2. I received an epidural while my Foley catheter was inside. - YES, NO

3. I received an epidural after my Foley catheter was removed. - YES, NO
